# The drive to strive: goal generation based on current needs

**DOI:** 10.3389/fnins.2013.00112

**Published:** 2013-06-27

**Authors:** Elisabeth A. Murray, Peter H. Rudebeck

**Affiliations:** Section on the Neurobiology of Learning and Memory, Laboratory of Neuropsychology, National Institute of Mental Health, National Institutes of HealthBethesda, MD, USA

**Keywords:** orbitofrontal cortex, macaque, reward, reinforcer devaluation, selective satiation, prefrontal cortex, anthropoid primates, amygdala

## Abstract

Hungry animals are influenced by a multitude of different factors when foraging for sustenance. Much of the work on animal foraging has focused on factors relating to the amount of time and energy animals expend searching for and harvesting foods. Models that emphasize such factors have been invaluable in determining when it is beneficial for an animal to search for pastures new. When foraging, however, animals also have to determine how to direct their search. For what food should they forage? There is no point searching for more of a particular food when you are sated from eating it. Here we review work in macaques and humans that has sought to reveal the neural circuits critical for determining the subjective value of different foods and associated objects in our environment and tracking this value over time. There is mounting evidence that a network composed of the orbitofrontal cortex (OFC), amygdala, and medial thalamus is critical for linking objects in the environment with food value and adjusting those valuations in real time based on current biological needs. Studies using temporary inactivation methods have revealed that the amygdala and OFC play distinct yet complementary roles in this valuation process. Such a network for determining the subjective value of different foods and, by extension, associated objects, must interact with systems that determine where and for how long to forage. Only by efficiently incorporating these two factors into their decisions will animals be able to achieve maximal fitness.

## Introduction

Animal learning theorists developed the selective satiation procedure to explore the behavioral effects of devaluing reinforcers (Colwill and Rescorla, 1985). Their purpose was to alter the value of what they call goals: the resources that animals strive to obtain in order to fulfill biological needs, such as foods, fluids, warmth, and other outcomes of behavior that often go by the term reinforcement or reward. By using the simple procedure of allowing an animal to consume one of two potential food rewards to satiety, the experimenter could manipulate the value of that reinforcer. Obviously, as an animal consumes one kind of food to satiety, and afterwards for a period of time, the value of that food decreases and animals will have less motivation to perform actions that will produce that “goal” or outcome. This tendency of an animal to avoid behaviors that produce a recently consumed resource in favor of some alternative is called a *devaluation effect*. With this laboratory procedure, researchers could classify an animal's behavior as “goal-directed,” meaning that the animal's choice depended on the behavioral outcome predicted to follow that choice, or, as a “habit,” meaning that the animal's choice did not depend on a predicted outcome. Other laboratory procedures, such as contingency degradation, serve the same ends (Hammond, 1980; Balleine and Dickinson, 1998).

Although these experimental procedures could hardly be more divorced from foraging behavior under natural conditions, their relevance should be immediately apparent. As animals forage in the wild, they need to make a multitude of decisions: about where to forage, for how long to forage, and about what items to expend energy to obtain in their immediate vicinity or at some more distant location. This applies to both the resources needed for nutrition and hydration, as well as others such as warmth, safety from predation, and procreation. Resources are not, however, stable over time in their abundance. A foraging animal has to take into account the rate of diminishing returns as it continues to forage, moving to a different location when the current rate of return drops. Animals therefore have to balance the energy expended foraging for food against the returns from doing so; such a problem is well-explained by the marginal value theorem (Charnov, 1976). Animals also must take into account their current state in order to make adaptive foraging decisions. There is no advantage, for example, in expending energy and risking predation in order to obtain more of a food when in a fully sated state.

Foraging, broadly defined, has many elements. First, animals have motivations and drives, that when fulfilled, satisfy their energetic or biological needs. For example, animals have hunger when undernourished. With a drive state in place, animals need to: (1) search for resources taking into account the time and energy needed for such a search; (2) identify resources, usually through vision, taste or smell; (3) predict the value of the available resources; and (4) select from among them. Finally, the animal consumes the resource. Between the drive state and consumption, then, least four steps can be considered separately. Here we consider two of them—valuation and selection—functions that are also relevant to understanding the neural bases of value-based decision making.

In this article, we will summarize two lines of investigation concerning resource valuation and selection in monkeys, with a focus on the effect of reinforcement devaluation. The first concerns what neural substrates and what kinds of information processing underlie devaluation effects in monkeys; the second concerns when devaluation occurs. Before addressing these topics, however, we briefly mention an advantage in studying resource devaluation in Old World monkeys.

**Figure 1:**
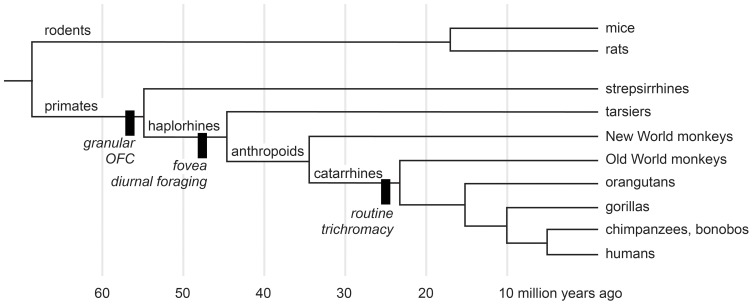
**A cladogram of primates and rodents on a linear time scale.** The black bars show innovations of selected lineages. Adapted from Passingham and Wise (2012) and O'Leary et al. (2013).

We study macaque monkeys, in part, because of their relatively close evolutionary relationship with humans. Nearly all animals forage, and all mammals certainly do. Despite the complexities of the urban jungle a trip to the market for a human is but a pale reflection of natural foraging behavior. Some people hunt, fish, and gather wild plant foods, but few people have depended on such foraging since the agricultural revolution of the Neolithic era. Modern people, however, descend from early humans, who were hunter-gatherers. So foraging is in our evolutionary history, and some of the mechanisms that subserved foraging decisions in our human ancestors were inherited from more distant ones. Figure 1 shows the evolutionary relationships most pertinent to the devaluation literature. According to the most recent morphological and molecular evidence (O'Leary et al., 2013), the lineages that eventually produced modern rodents and primates diverged nearly 65 million years ago, at about the same time as the extinction of dinosaurs. The evidence from comparative neuroanatomy shows that a key cortical region, consisting of granular and dysgranular parts of the orbitofrontal cortex (OFC), first emerged in early primates (Preuss and Goldman-Rakic, 1991). All primates, including Old World monkeys and humans, share this region through inheritance from those common ancestors (Figure 1). As the evidence reviewed in the next two sections indicates, the OFC, together with subcortical structures, subserves resource valuation in macaque monkeys. If we are to understand the mechanisms of resource valuation in humans—an endeavor roughly corresponding to the field called neuroeconomics—Old World monkeys provide a more closely related starting point than other common laboratory animals.

## What structures, what information subserve reinforcer devaluation?

Many behavioral tasks have been used as models of foraging behavior in macaques. For example, tasks have assessed the ability to take into account delayed (Hayden and Platt, 2007; Kim et al., 2008), effortful (Walton et al., 2006) or probabilistic outcomes (Amiez et al., 2006; Kennerley et al., 2006; Rudebeck et al., 2008), to track changes in reward probability or amount over time (Sugrue et al., 2004; Yang and Shadlen, 2007; Pearson et al., 2009; Noonan et al., 2010; Walton et al., 2010), to integrate costs and benefits (Roesch and Olson, 2004; Kennerley et al., 2009; Seo and Lee, 2009; Amemori and Graybiel, 2012), to switch away from simulated patches of resources that decline over time (Agetsuma, 1999; Hayden et al., 2011), and so on. All these tasks have increased our understanding of brain mechanisms that may have arisen to guide foraging decisions. In this review we consider findings from experimental studies that manipulate satiety as a way to alter—typically to lower—the value of food.

There are several reasons to consider studies that alter the state of satiety, and to consider them in isolation. First, varying the level of satiety provides a window on how value is represented, updated and used to guide foraging decisions. Second, although object-reward associations are a common feature of laboratory-based foraging tasks, recent evidence indicates that different types of object-reward associations are mediated by different sets of brain circuits. For example, in macaques, different brain structures support stimulus-reward association as measured by linking objects with reward contingency vs. linking objects with food value (Rudebeck and Murray, 2011). Third, satiety is an independent variable that can be measured in its own right. Foods used in an experiment can be controlled in terms of weight, flavor, caloric content, etc. In addition, experimenters can control the animal's motivation for a particular food, by feeding an animal only that type of food in a selective satiation procedure, or generally, by feeding daily rations. As indicated earlier, in this review we focus on studies using sensory-specific satiety as a means of manipulating food value. Studies employing selective satiety typically isolate the valuation and selection steps of foraging decisions from other factors such as navigation and memory for choice history. Although these latter functions are indeed essential features of naturalistic foraging, a simplified method for evaluating foraging decisions will facilitate experimental investigation of the underlying neural substrates.

### Decisions among objects

Many of the studies we review have relied on the devaluation test developed for macaques (Malkova et al., 1997). The devaluation test is often carried out in two stages: training and test. In the training phase monkeys are familiarized with a large number of objects and their associated food rewards. To achieve this, monkeys learn 60 discrimination problems concurrently. Just as in standard discrimination learning problems, on each trial, a pair of objects consisting of one S+ (baited) and one S− (unbaited) is presented for choice. Unlike the standard procedure, however, half of the S+ objects are rewarded with one kind of food, designated food 1, and half are rewarded with a different food, designated food 2. Through trial and error, monkeys learn to choose the S+ objects to obtain the food reward hidden underneath. In the test phase, which consists of a series of probe tests, only the S+ objects are used; now, for the first time, monkeys are offered the choice between food-1 and food-2 associated objects. Importantly, in the test phase, novel combinations of objects are used on each trial. There is no “wrong” answer; each object overlies the food with which it was associated in the training phase.

Three different conditions are employed in the test phase. One condition serves to measure baseline choices of food-1 and food-2 associated objects. Monkeys are simply allowed to choose and displace objects to obtain the food reward hidden underneath. A second condition involves probe tests conducted after prefeeding (selective satiation) with food 1, and yet a third condition involves probe tests conducted after prefeeding with food 2. In theory, prefeeding serves to temporarily devalue the food. Thus, the probe tests conducted after feeding to satiety reveal the ability of monkeys to link objects with current, updated food value. A critical element of the probe tests is that choices are made without any additional opportunity to learn about the object–food value associations; optimal choices can only occur if monkeys automatically link objects with the new food value, and use that information to guide choices.

After being sated on one food, intact monkeys spontaneously shift their choices away from the objects overlying the devalued food in favor of objects overlying the higher valued (non-sated) food; in other words, they show robust devaluation effects. This can be quantified by calculating a “difference score,” which reflects the extent to which monkey's object choices on the probe tests conducted after prefeeding differ from the baseline condition (no prefeeding). Importantly, the devaluation effects reveal that monkeys care about the value of the foods that result from their object choices.

Although the task used in monkeys is based on devaluation paradigms first developed and used with rats (Colwill and Rescorla, 1985; Balleine et al., 1994; Hatfield et al., 1996), several changes were made to make the task better suited to macaques. First and foremost, monkeys were required to select objects, on the basis of vision, and to displace those objects to obtain food reward hidden underneath, behaviors that emerge with little or no training. Thus, in experiments with monkeys, object choices replace the food cup approach and lever press responses typically used with rats. A second change relates to how the probe tests are administered. If food reward is provided during the probe test, which is meant to assess animals' expectancy of reward value, it may lead to relearning of cue or action-value associations, thereby making the test less sensitive. Accordingly, in tasks with rodents, probes tests are typically carried out under extinction, i.e., in the absence of food reward. By contrast, the tests that assessed monkeys' abilities to link objects with food value were not conducted in extinction. To prevent cue-value learning, however, and to maximize the number of trials per test, the probe tests in monkeys used different pairs of objects on each trial. If within-trial learning about particular objects occurred, it could not aid the monkeys on subsequent trials. In support of this supposition, performance of lesion groups tends to be stable across test sessions (Izquierdo and Murray, 2010).

Using the object-based task described above, several studies in monkeys have investigated the neural bases of reinforcer devaluation effects. In the following sections we summarize the results from such studies and explore their implications.

#### Amygdala

**Figure 2:**
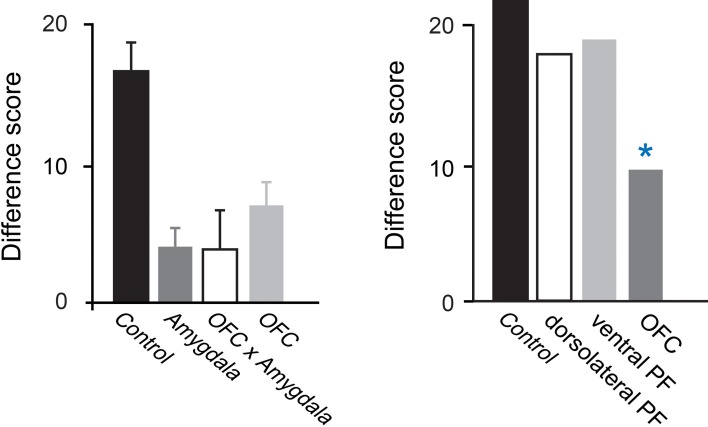
**Devaluation effects for object–outcome associations.** Summary of the scores of several groups of monkeys studied using the devaluation task. The scores for each group are reflected in a single bar; a higher score indicates greater sensitivity to changes in food value. Intact monkeys avoid choosing objects associated with a devalued food, as indicated by the high difference scores obtained by the unoperated control group. In the **left panel**, monkeys with bilateral lesions of either OFC or the amygdala continue to choose much like they had before the selective satiation procedure, as reflected in the low difference scores. In addition, monkeys with a surgical disconnection that prevents the intrahemispheric interaction of the amygdala and OFC cannot efficiently link objects with the current value of a food reward. Although the operated groups were subjects in different studies, all groups were tested in a similar fashion and had similar experimental histories. In the **right panel**, scores of groups with lesions in different sectors of prefrontal cortex are provided. ^*^p < 0.05. Data in left panel are from Baxter et al. (2000), Izquierdo et al. (2004), and Izquierdo and Murray (2007). Data in the right panel are from Baxter et al. (2008, 2009). Modified from Murray and Wise (2010). Abbreviations: Control, unoperated control monkeys; Amygdala, monkeys with bilateral excitotoxic amygdala lesions; OFC, monkeys with bilateral aspiration lesions of the orbital prefrontal cortex; OFC × Amygdala, monkeys with surgical crossed disconnection of the amygdala and OFC; dorsolateral PF, monkeys with bilateral aspiration lesions of the dorsolateral prefrontal cortex; ventral PF, monkeys with bilateral aspiration lesions of the ventral prefrontal cortex.

Studies examining the effects of excitotoxic amygdala lesions have revealed that the macaque amygdala is an essential part of the circuit underlying devaluation effects (Malkova et al., 1997; Izquierdo and Murray, 2007; Machado and Bachevalier, 2007). In Figure 2, the higher the difference score the greater the sensitivity to reinforcer devaluation. For example, a difference score of 20 indicates that monkeys shifted their choices toward the object covering the high value (non-sated) food on 67% of trials (20 out of a possible 30). Monkeys with selective, bilateral lesions of the amygdala fail to choose objects appropriately after changes in food value. As shown in Figure 2, left panel, relative to controls, monkeys with amygdala lesions exhibit a significantly lower tendency to choose objects overlying the high value (non-sated) food, and this deficit is long lasting (Izquierdo and Murray, 2007).

The results of additional tests have helped illuminate the nature of the impairment. First, because monkeys with amygdala lesions learn visual discrimination problems at a normal rate (Malkova et al., 1997; Izquierdo and Murray, 2007; Machado and Bachevalier, 2007), we can rule out global changes in visual perception as a cause for the impairment. In addition, because monkeys with amygdala lesions have intact satiety mechanisms and because the effect of satiety transfers from the home cage to the test apparatus, we can conclude that the impairment does not result from altered satiety mechanisms or from an inability to discriminate foods. Importantly, when considering choices among palatable foods, there is little or no evidence that monkeys with amygdala damage have altered food preferences (Aggleton and Passingham, 1981; Murray et al., 1996; Izquierdo and Murray, 2007; Machado and Bachevalier, 2007; cf. Agustín-Pavón et al., 2011) or altered motivation to work for food (Baxter et al., 2000). Indeed in test sessions not preceded by satiation monkeys with amygdala lesions continue to make object choices in line with their subjective preferences. Thus, it appears that the disruption of devaluation effects after amygdala lesions is specifically due to a failure to link objects with the updated value of the food reward or, alternatively, a difficulty in using this information to guide object choices.

#### OFC

Figure 2 shows that, like monkeys with bilateral amygdala lesions, monkeys with bilateral aspiration lesions of the OFC exhibit significantly reduced devaluation effects (Izquierdo et al., 2004; Machado and Bachevalier, 2007; Baxter et al., 2009). The left panel of Figure 2 shows results from monkeys that were tested in a manual test apparatus [group OFC; Izquierdo et al. (2004)], whereas the right panel shows results from monkeys that were tested in an automated apparatus [group OFC; Baxter et al. (2009)]. Comparable results were obtained across studies; in each case, monkeys with aspiration lesions of OFC were insensitive to changes in food value. As was the case for the amygdala, low level accounts for the impairment are extremely unlikely; explanations of the impairment after OFC lesions in terms of alterations in visual perceptual processes, food preferences, and willingness to work for food rewards can be ruled out (Izquierdo et al., 2004).

The OFC in macaques has been found to consist of over a dozen cytoarchitectonic fields (Carmichael and Price, 1994) that, based on patterns of anatomical connections, have been grouped into two distinct networks (Carmichael and Price, 1996; Saleem et al., 2008). Within OFC, lateral areas correspond to a “sensory network” and more medial areas correspond to a “visceromotor network.”

Given these putative networks, investigators have studied whether there is evidence for dissociation of function. Relevant to the present discussion, it has been found that the lateral part of OFC corresponding to areas 11 and 13, but not the medial part of OFC corresponding to areas 14 and 10 m, is essential for devaluation effects (Rudebeck and Murray, 2011). Notably, the impairment after lesions restricted to areas 11 and 13 was found using selective, excitotoxic lesions, confirming that cell loss in this area yields the behavioral impairment. In addition, the magnitude of the impairment matched that seen after aspiration lesions of the whole OFC (Izquierdo et al., 2004). Taken together, these findings suggest that damage to areas 11 and 13 is responsible for the entire effect.

#### Medial MD thalamus

The medial or magnocellular division of the mediodorsal nucleus of the thalamus (MD) is heavily interconnected with both the OFC and the amygdala in macaques (Porrino et al., 1981; Goldman-Rakic and Porrino, 1985). Accordingly, this region's contribution to value-based decision making is of interest. Mitchell and colleagues found that bilateral lesions of the medial portion of MD disrupted devaluation effects (Mitchell et al., 2007). Thus, medial MD thalamus is a part of the circuitry contributing to adaptive choices after changes in food value.

The pattern of connections of the medial MD thalamus raises the possibility that it contributes more to value-based decision making than currently appreciated. There are at least three aspects of its connectivity that point to a significant contribution. First, the medial MD thalamus is not only reciprocally connected with OFC, but at least some of the projections cross to the other hemisphere (Preuss and Goldman-Rakic, 1987). Second, there are fairly widespread projections from medial MD thalamus to the frontal cortex (Ray and Price, 1993). For example, thalamocortical projections arising in medial MD reach dorsal and ventral sectors of lateral prefrontal cortex in addition to medial and orbital frontal regions. Third, unlike the sensory relay nuclei of the thalamus, medial MD receives inputs from several parts of the forebrain (e.g., amygdala, entorhinal cortex, and perirhinal cortex) that project directly to the frontal cortex (Russchen et al., 1987; Ray and Price, 1993). Moreover, medial MD receives projections from parts of the basal forebrain, specifically substantia innominata and ventral pallidum (Russchen et al., 1987). These structures have enigmatic functions, but there is evidence suggesting that they contribute to value-based decision making (Tachibana and Hikosaka, 2012). Thus, several forebrain structures, including basal forebrain, amygdala and ento- and perirhinal cortex, project to medial MD and might—via thalamocortical projections arising in medial MD—influence prefrontal cortex function.

#### Amygdala, OFC, thalamic interactions

A few studies have considered whether a given set of structures needs to interact in guiding value-based decision making, as assessed by the devaluation task. In one study, monkeys received a unilateral lesion of the amygdala in one hemisphere and a unilateral lesion of the OFC in the other hemisphere, together with section of the forebrain commissures. The OFC and amygdala are reciprocally interconnected (Carmichael and Price, 1995a; Ghashghaei et al., 2007) and this surgical preparation (a so-called disconnection surgery) prevents the functional interaction of the amygdala and OFC. Monkeys with the amygdala-OFC surgical disconnection were tested on the devaluation task, and found to be impaired (Baxter et al., 2000). In addition, Izquierdo and colleagues (2010) tested whether the medial MD thalamus needs to functionally interact with other brain regions in mediating devaluation effects. In their experiment, monkeys with unilateral lesions of the amygdala and OFC in one hemisphere received excitotoxic lesions of medial MD thalamus or nucleus accumbens in the other hemisphere. Like MD, the nucleus accumbens—a part of the ventral striatum—is interconnected with both the OFC and amygdala (Yeterian and Van Hoesen, 1978; Russchen et al., 1985; Haber et al., 2006). Using this crossed disconnection design, it was found that medial MD thalamus but not nucleus accumbens was an essential part of the circuitry allowing monkeys to make adaptive responses to changes in food value. Thus, in normal circumstances, the amygdala, medial MD thalamus and OFC work together in guiding choices based on the current biological value of different foods.

#### Non-essential structures

Several brain regions are not essential for devaluation effects in monkeys, at least not as assessed using the object-based task described above. Selective lesions of either the hippocampal formation or the perirhinal cortex fail to disrupt devaluation effects (Machado and Bachevalier, 2007; Chudasama et al., 2008). Because these structures are adjacent to the amygdala, the negative results indicate that the behavioral effects of amygdala damage are unlikely to be due to inadvertent damage to regions outside the amygdala, and reinforce the notion that, within the temporal lobe, only the amygdala is essential for mediating devaluation effects. Regions of prefrontal cortex outside the OFC have also been examined for their contributions to devaluation effects. Neither the ventral prefrontal cortex, situated just lateral to the OFC, nor the dorsolateral prefrontal cortex are essential for devaluation effects (Baxter et al., 2008, 2009). Likewise, the anterior cingulate cortex (area 24), situated on the medial surface of the hemisphere and which is also interconnected with the amygdala (Van Hoesen et al., 1993), is not essential for devaluation effects (Chudasama et al., 2012). Finally, as indicated earlier, only the lateral portion of OFC, the part corresponding to the “sensory,” as opposed to the medial OFC “visceromotor” network, of Price and colleagues, is essential for devaluation effects (Rudebeck and Murray, 2011).

#### Summary of object-outcome devaluation studies

The devaluation task is a tool that permits insight into the components of value-based decision making; it interrogates monkeys' abilities to link objects with particular properties of food rewards, including not only their sensory properties but also their value. In a series of probe trials, monkeys report on their estimation of the value of expected outcomes of their object choices. A substantial number of studies now implicate a circuit including the lateral orbital cortex areas 11 and 13, basolateral amygdala, and medial, magnocellular MD thalamus in helping monkeys make adaptive choices. These structures are needed when monkeys are required to make visually guided object choices on the basis of subjective value associated with a specific food. Under conditions of stable object-value associations, these structures are not needed to make appropriate choices, presumably because “value” can be processed, represented, and stored in many places in the brain. The role of these structures in adaptive valuation of specific outcomes may be related to model-based reinforcement learning mechanisms (Sutton and Barto, 1998). Work in rats has shown that the OFC is critical for model-based, as opposed to model-free, reinforcement learning (Takahashi et al., 2009; McDannald et al., 2011; Jones et al., 2012).

Despite advances in identifying this network of areas, one outstanding question is what structures interact with the OFC, amygdala and MD to allow the updating of food value. Deficits following OFC or amygdala lesions only affect the choice of objects or actions associated with food rewards, not the appreciation, discrimination, or selection of food rewards themselves (Izquierdo et al., 2004; Izquierdo and Murray, 2007). It is known that parts of the hypothalamus in macaques are involved in satiety mechanisms (Rolls et al., 1986) but it is unclear how such parts of the brain interact with OFC and amygdala to achieve the updating of object value. Expanding our understanding of how these areas interact during feeding and satiety in macaques will be a rich avenue for future research.

### Decisions among actions

The foregoing studies assessed the ability of monkeys to link objects with the value of outcomes. Although it is clear that monkeys are making choices based on the value of the expected outcome, namely, the value of the food that will be obtained by choosing a particular object, there are some unresolved issues. One major question surrounds the nature of the association underlying the devaluation effects. Early work examined the effects of reinforcer devaluation in the context of *action-outcome* associations, as opposed to *object-outcome* associations. Indeed, devaluation tasks have been theorized to interrogate knowledge regarding the causal relationships between actions and the outcomes to which they lead (Dickinson and Balleine, 1994). To evaluate whether the neural circuitry underlying object-outcome associations in macaques would hold for action-outcome associations, we devised an action-outcome task for macaques and then assessed the involvement of the amygdala and OFC on this new task (Rhodes and Murray, 2013).

Although several studies have examined the role of frontal cortex in the learning and performance of action-reward associations in monkeys, they have often involved reversal learning or matching tasks (Hadland et al., 2003; Kennerley et al., 2006; Rudebeck et al., 2008; Chudasama et al., 2012). With one exception (Chudasama et al., 2012), no studies have applied a direct test of whether behavior is influenced by the current value of the goal, as in the case of a devaluation task. In the new task, monkeys were required to make two different responses for two different food rewards. The actions, “tap” and “hold,” were two mutually exclusive responses performed via manual contact with a touch-sensitive screen. A *tap* response consisted of six rapid touches to the screen, all performed within 2 s, and a *hold* response consisted of maintained contact with the screen for 2 s. Once the individual actions were learned, monkeys were given a choice between performing either the tap or the hold response on every trial to earn the corresponding food reward.

**Figure 3:**
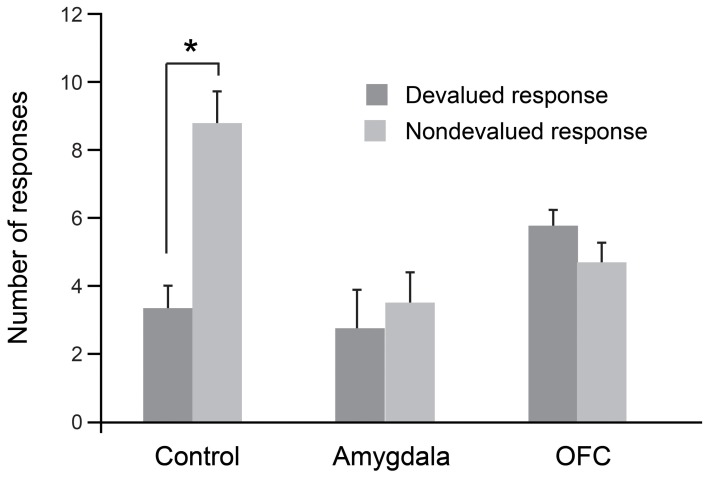
**Devaluation effects for action–outcome associations.** After selective satiation, unoperated control monkeys perform significantly more of the responses associated with the higher value (non-devalued) food relative to responses associated with the lower value (devalued) food. Thus, controls show classic devaluation effects. Monkeys with either bilateral excitotoxic lesions of the amygdala or bilateral excitotoxic lesions of the OFC fail to show devaluation effects. ^*^p < 0.05. Modified from Rhodes and Murray (2013).

As was the case for the object-based task, we evaluated each monkey's response preference after sating the monkey on one of the two foods. In this procedure, monkeys were allowed to consume one of the two foods to satiety and were then tested for tap/hold preference under extinction. As shown in Figure 3, when monkeys were tested for their willingness to perform actions associated with either the higher-value (non-devalued) or lower-value (devalued) food, unoperated controls performed significantly more of the responses associated with the non-devalued food. As predicted from results of studies with rats (Johnson et al., 2009), and as was the case for the object-based studies described earlier, bilateral selective, excitotoxic lesions of the amygdala disrupted devaluation effects. Monkeys with amygdala lesions also made significantly more omissions relative to controls; this accounts for the overall lower number of responses in this group (Figure 3). In addition, counter to findings in rats (Ostlund and Balleine, 2007), bilateral, selective excitotoxic lesions of OFC likewise disrupted devaluation effects (Rhodes and Murray, 2013). Taken together with the data from object-based tasks, these results strongly implicate both the amygdala and OFC in value-based decision making.

### Decisions based on internal context

Although we and others have interpreted these data in the framework of value updating, an alternative possibility is that the amygdala is critical for either representing internal context or using internal context to guide behavior. On this view, amygdala damage disrupts so-called devaluation effects because of its influence on processing of internal context, which is needed for the “value-updating” function. The devaluation task does not allow us to discriminate between the two possible roles for the amygdala. If the amygdala plays a general role in guiding choice behavior with respect to internal context, then amygdala damage should cause impairments in other situations where object choices are based on internal context, and not just when the choices are based on updating of the value of food associated with the objects.

**Figure 4:**
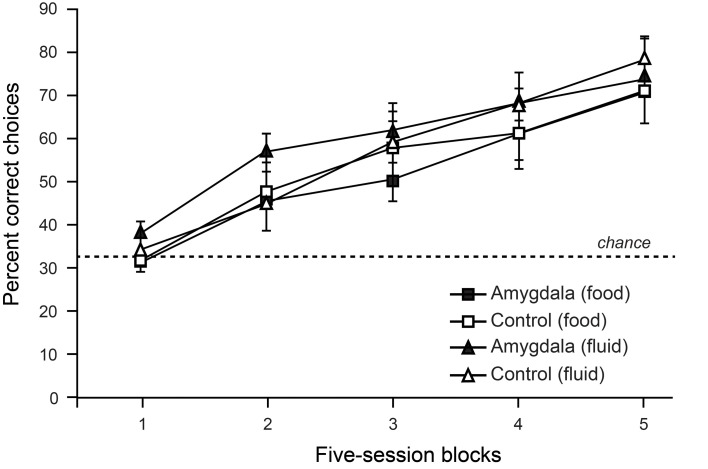
**Choices between objects associated with food vs. fluid resources.** On each trial, monkeys were allowed to choose among objects associated with food or fluid reward and a third, nonreinforced object. Only objects associated with food reward were baited when monkeys were hungry, and only those objects associated with fluid were baited when monkeys were thirsty. Curves show scores for sessions involving object choices when monkeys were either hungry (food) or thirsty (fluid). Amygdala lesions had no effect on learning rates in this task. Modified from Rhodes et al. (2012).

To test the contribution of the amygdala to object choices based on internal context we trained monkeys on a discrimination whereby objects associated with food (but not water) were baited when the monkey was hungry, and objects associated with water (but not food) were baited when the monkey was thirsty. To solve this task monkeys were required to choose objects yielding the reward congruent with their internal motivational state. As shown in Figure 4, lesions of the amygdala failed to disrupt either learning or performance of this task (Rhodes et al., 2012). Accordingly, it seems unlikely that the involvement of the amygdala in devaluation tasks, which depends in part on a change in internal context, is due to the amygdala playing a general role in representing, or using, internal context.

## When does devaluation occur?

### Amygdala

Given the results from selective excitotoxic lesions cited above, the deficit on the devaluation task could result from a disruption in either or both of two mechanisms: (1) updating the value of the food during selective satiation; or (2) linking objects with the current value of a food to guide object choices. Using temporary inactivation techniques, Wellman and colleagues (2005) have provided a significant advance in understanding the amygdala's contribution to devaluation effects. Monkeys were trained on the devaluation task as described above. Wellman and colleagues then infused the GABA agonist muscimol into the basolateral amygdala, bilaterally, either before or after the selective satiation procedure, thereby producing a temporary inactivation of the basolateral amygdala. Finally, they administered the probes tests in the usual manner. Importantly, infusions *before* selective satiation would lead to inactivation during the selective satiation procedure, which is when the value-updating process is thought to occur, as well as during the probe tests, when object choices are made. By contrast, infusions *after* the selective satiation procedure would affect amygdala activity only during the choice tests. Wellman et al. found that temporary inactivation of the amygdala during the selective satiation procedure, but not afterward, disrupted the devaluation effects (Wellman et al., 2005). This finding shows that the amygdala is essential specifically for the value-updating process; once the new value is registered, the amygdala is no longer needed.

### OFC

As described above for the amygdala, to dissect the contributions of OFC to different phases of the devaluation task, West and colleagues examined the effects of inactivation of OFC before and after the selective satiation procedure (West et al., 2011). They found that inactivation of OFC either before or after the selective satiation procedure disrupted devaluation effects. In this experimental design, the interpretation of the results is complicated by the fact that, in both conditions, OFC is inactive during the probe tests. Accordingly, there are two possible interpretations of the pattern of results. One possibility is that OFC is essential for both the value-updating function and the selection function. Alternatively, however, it is possible that OFC is essential for only the selection function. Some preliminary data from our laboratory may inform this debate. To explore the potential independent contributions of areas 11 and 13 to devaluation effects, Howland and colleagues (2011) examined the effects of transient inactivations of each of these regions separately on monkeys' performance on the devaluation task. Inactivation of neurons in area 13 during the selective satiation procedure disrupted devaluation effects; by contrast, inactivation after selective satiation but before choice tests had no effect (Howland et al., 2011). Inactivation of area 11 produced the converse result; inactivation of area 11 during the selective satiation procedure had no effect whereas inactivation after selective satiation disrupted devaluation effects. In separate sessions, carried out as a control, inactivation of area 11 had no effect on performance of familiar discrimination problems. Accordingly, area 11 is not necessary for selection of objects generally, but only in conditions when a new value must be accessed. These preliminary findings suggest that area 13 is essential for encoding the change in reward value that occurs during selective satiation but not for longer-term storage of that value. Area 11, by contrast, appears to be essential for storage and/or retrieval of the updated value but not for the value-updating function. Such differential contributions of parts of OFC to value-updating may be related to the relative strength of connections, either reciprocal or non-reciprocal, with the amygdala across the OFC (Carmichael and Price, 1995a; Ghashghaei et al., 2007). Thus, discrete subregions of OFC may make selective contributions to the different phases of the devaluation task.

In summary, recent work with reversible inactivation techniques indicates that the basolateral amygdala and lateral OFC have dissociable roles in mediating devaluation effects. The basolateral amygdala is required for updating the value of a food reward during feeding. Once that change has been registered, the amygdala is no longer needed to guide adaptive choices. The OFC appears to be important for two functions: (1) updating and registering a change in value and (2) storing and/or retrieving updated value to guide object choices. Future studies will need to assess whether other subdivisions within OFC or other regions of prefrontal cortex outside OFC might also contribute to the storage and retrieval of updated values.

## Comparison of satiety mechanisms in monkeys and humans

A large body of work has revealed that the activity of neurons in both the OFC and amygdala in monkeys is modulated by appetitive rewards, such as fruit juice, and stimuli that predict them (Niki and Watanabe, 1979; Sanghera et al., 1979; Thorpe et al., 1983; Tremblay and Schultz, 1999; Sugase-Miyamoto and Richmond, 2005; Padoa-Schioppa and Assad, 2006). More fine-grained experimental methods have allowed researchers to show that these reward-related responses are not simply related to the salience or attentional nature of such rewards. Instead, neuronal firing rates within the OFC and amygdala, as opposed to other parts of the brain such as the premotor or parietal cortex, reflect subjective value (Roesch and Olson, 2004; Paton et al., 2006; Morrison and Salzman, 2009; Leathers and Olson, 2012). Notably, neurons in OFC signal not only the value of received rewards, but also the value of expected rewards. During cue presentation, for example, the activity of neurons in OFC reflects the value of the reward predicted by that cue, largely independently of cue identity and location of any upcoming response (Thorpe et al., 1983; Wallis and Miller, 2003; Kennerley and Wallis, 2009; Luk and Wallis, 2013). Furthermore, in the context of a task in which visual stimuli are paired with specific appetitive (fluid) or aversive (air puff) outcomes through Pavlovian conditioning, neurons in OFC signal expected outcomes with a shorter latency than do amygdala neurons (Morrison and Salzman, 2011). Moreover, OFC neurons signal expected value largely independently of whatever alternative outcomes might be available at the same time (Padoa-Schioppa and Assad, 2008) This reward expectancy signal may mediate, at least in part, the devaluation effects described above.

Also relevant to devaluation, neurophysiological studies have reported satiety-dependent changes in neuronal activity within OFC (Critchley and Rolls, 1996; Bouret and Richmond, 2010). Specifically, value responsive neurons in OFC exhibit reductions in firing rate as monkeys become increasingly sated. It should be noted, however, that satiety-dependent changes in firing rate have also been reported in parts of the prefrontal cortex outside of those essential for devaluation, including Walker's areas 14 and 12 (Rolls et al., 1989; Critchley and Rolls, 1996; Bouret and Richmond, 2010).

A number of investigations have started to uncover the neural substrates of satiety and devaluation effects in humans (O'Doherty et al., 2000; Small et al., 2001; Gottfried et al., 2003; Kringelbach et al., 2003). In the first study of its kind, O'Doherty and colleagues scanned human volunteers while they were presented with distinct visual stimuli paired with either banana or vanilla odors. After this initial scanning session, volunteers ate bananas until they were sated and then were scanned again in the same stimulus-odor task (O'Doherty et al., 2000). Mirroring the findings from macaque monkeys, parts of the OFC showed decreased activation to banana odors following satiation. Activation of OFC in response to the non-sated vanilla odor, by contrast, was not altered. Decreased activation in OFC to visual stimuli associated with sated foods has been reported for different types of foods, such as chocolate or food-related liquids, such as tomato juice (Small et al., 2001; Kringelbach et al., 2003). An additional study by Gottfried and colleagues extended these findings by showing that the neural circuitry involved in devaluation in humans includes parts of both the OFC and the amygdala (Gottfried et al., 2003). Again, this complements the work in macaques (Baxter et al., 2000), which has revealed that interaction between the OFC and amygdala is critical for the ability to update the value of food outcomes. In summary, the available work in humans is largely in agreement with the reports from macaque monkeys. One avenue for future research in both monkeys and humans will be to explore the role of different parts of the OFC in representing and updating stimulus-value associations.

## Putting it all together

**Figure 5:**
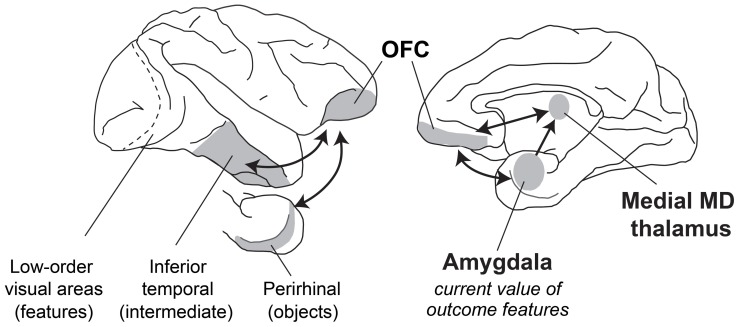
**A schematic of brain structures involved in devaluation.** Lateral (left) and medial (right) views of the macaque brain showing locations of structures involved in devaluation. Visual information from the retina is processed in primary visual cortex and then in a series of cortical fields terminating in inferior temporal cortex. More rostral regions of inferior temporal cortex, including the multimodal perirhinal cortex, represent more complex conjunctions of features, such as objects. This visual information signaling object identity is then combined with features from other sensory modalities (e.g., gustatory, olfactory, visceral sensory inputs) in the granular OFC. Through interaction of the amygdala with MD thalamus and granular OFC, these sensory representations of objects and foods can be linked with value, based on the current biological needs of the animal.

When evaluating what resources to forage for in their environment, how do primates make appropriate choices? Typically, the first step involves visual sensory processing to identify objects and foods in the environment. In macaques, this function is carried out in the inferior temporal cortex “object analyzer” pathway, which we take to include the multimodal perirhinal cortex (Figure 5). At the same time, gustatory, olfactory, visceral and tactile information comes together in caudal, agranular OFC (Pritchard et al., 1986; Morecraft et al., 1992; Barbas, 1993; Carmichael et al., 1994; Carmichael and Price, 1995b), which likely houses representations of the flavor, texture, and palatability of foods. Next, both perirhinal cortex and agranular OFC project to the granular OFC, where these two streams of sensory information converge (Carmichael and Price, 1995a; Cavada et al., 2000; Saleem et al., 2008); granular OFC appears to be the earliest site in OFC where visual sensory information regarding objects may be linked to the sensory properties of foods. Finally, through interaction with the amygdala and medial MD as well as parts of the hypothalamus, sensory information about potential resources in the environment is ascribed value, based on the current state of the animal. With information about the identity and value of potential foods in their environment at their disposal, monkeys can then decide which resources or foods are worth pursuing. Of course the picture is more complicated than this. We have provided a simplified picture of the brain structures and interactions between them that might take place.

But how does the OFC–amygdala–MD thalamus circuit influence action? To contribute to foraging, these structures must affect the motor system directly or indirectly. Recent neurophysiological evidence suggests how the OFC–amygdala–MD circuit influences processing in visuomotor pathways that plan movements, through a mechanism akin to top–down attention and biased competition (Pastor-Bernier and Cisek, 2011). These influences are probably mediated by connections between OFC and ventral, medial and dorsolateral prefrontal cortex (Barbas and Pandya, 1989; Carmichael and Price, 1996), which contribute to multisynaptic pathways to dorsal premotor areas, mainly via dorsal and dorsolateral prefrontal cortex (Takahara et al., 2012), and cingulate premotor areas, mainly via medial prefrontal cortex. In addition, the amygdala projects to premotor cortex directly (Avendano et al., 1983; Morecraft et al., 2007). Thus, both OFC and the amygdala are well-situated to influence the targets selected for action.

## Conclusions

Considered together, the research reviewed in this article leads to several conclusions. One set of conclusions regards what brain structures and what kinds of computations underlie foraging decisions based on updated outcome valuations. First, the evidence points to functional interactions among at least the OFC, the amygdala and the magnocellular or medial MD thalamus as the neural substrates for reinforcer devaluation based on selective satiation (Figure 5). Second, it indicates that the key computations involve the establishment of conjunctive representations between certain sensory features of reinforcing outcomes—potentially through interaction with agranular OFC—and an updated valuation of those features. Third, the computations performed by these OFC–amygdala circuits do not depend on whether the monkey makes a decision among objects or a decision among actions. These results provide further support for the idea that the key computation involves establishing a conjunction between certain sensory properties of the outcome and their current biological value. Fourth, the devaluation effect does not depend on reading out the internal state of the animal *per se*. If it did, the amygdala would play a necessary role in choosing between objects that will produce food when hungry and fluid when thirsty. We found that this is not the case.

A second set of conclusions concerns when the devaluation computations occur. The key results come from studying neural inactivation during the consumption of a given kind of food, as opposed to inactivations afterward, when monkeys make these decisions. First, amygdala function is necessary as the animal consumes the food. Although we have no direct evidence on a fine time scale, we suspect that each ingestion event causes a small devaluation of a given food until it reaches a floor, which is called selective satiation. Second, the lateral OFC needs to function during both the consumption/satiation phase and later, when the animals make their decisions. Third, preliminary evidence suggests that different parts of the OFC perform specialized functions in this regard: the more rostral part, area 11, needs to function when the animal uses the updated conjunctions to choose among objects; the more caudal part, area 13, like the amygdala, must perform its function as satiation progresses.

From a larger perspective, we can appreciate that the selective satiation procedure provides more than a tool for classifying behavior as “goal-directed” or “habits.” It provides a window on the computations underlying the decisions that primates and other animals need to make during natural foraging. No foraging decision would serve an animal's needs if it failed to take into account the animal's current state. As a result, the level of satiation or deprivation on a vast array of necessary resources enters into the computation underlying all foraging decisions.

How then, do we put these conclusions together with those concerning the role of OFC in linking objects (or choices among objects) with outcomes (Rudebeck et al., 2008; Noonan et al., 2010; Walton et al., 2010; Rushworth et al., 2011)? Studies of probabilistic reinforcement have revealed a role for OFC in a kind of updating function that differs from the one emphasized in this article (Walton et al., 2010). That kind of updating reflects changes in the likelihood of reward or contingency. As the likelihood of reward changes over time, the lateral parts of OFC update the representations of objects to reflect those changes. Accordingly, we term this *object-outcome updating*. In this article, we have stressed a different updating function, also mediated by the lateral parts of OFC, which might be termed *outcome-value updating*. Whereas the studies of probabilistic reinforcement emphasize the conjunctive representations of objects and outcomes, the studies using reinforcer devaluation emphasized here involve conjunctive representations of current biological value with a feature (or features) of those outcomes. The object–outcome updating goes hand in hand with the updating involved in representing the current biological value associated with features of an outcome. We view these different kinds of updating as complementary; it is possible that they depend on different circuits or populations of neurons that are present in the OFC (O'Neill and Schultz, 2010). For food resources, for example, *object–outcome updating* keeps current information about the likelihood of food in the environment; the *outcome–value updating* speaks to the sensory properties of the predicted outcome and incorporates the animal's current state into these representations, which reflects its current biological needs. Both of these types of updating are critical to a foraging animal: one relates to the external world and the likelihood of food, the other to the animal's internal needs and desires. Efficiently and adaptively incorporating both to guide foraging will help an animal attain maximal fitness.

### Conflict of interest statement

The authors declare that the research was conducted in the absence of any commercial or financial relationships that could be construed as a potential conflict of interest.
